# Antibiotic Residues in Muscle Tissues of Lueyang Black-Bone Chickens Under Free-Range Mountainous Conditions and Their Association with Gut Microbiota

**DOI:** 10.3390/microorganisms13102239

**Published:** 2025-09-24

**Authors:** Mingming Zhao, Shuang Zeng, Linqing Shao, Ling Wang, Tao Zhang, Hongzhao Lu, Wenxian Zeng

**Affiliations:** 1 School of Biological Science and Engineering, Shaanxi University of Technology, Hanzhong 723001, China; 2 Shaanxi University Engineering Research Center of Quality Improvement and Safety Control of Qinba Special Meat Products, Hanzhong 723001, China; 3 Qinba State Key Laboratory of Biological Resources and Ecological Environment, Shaanxi University of Technology, Hanzhong 723001, China

**Keywords:** antibiotic, gut microbiota, metabolomic, microbiome, muscle tissue, analysis of correlation

## Abstract

The absorption, transport, and distribution of antibiotics in animals are influenced by the composition and function of the intestinal microbial community. However, most existing studies have focused on intensive farming systems involving the artificial addition of antibiotics. For free-range local chicken breeds in mountainous areas without antibiotic additives, systematic research on the presence of antibiotic residues in their muscle tissues and their association with the gut microbiota is lacking. Therefore, in this study, mountainous free-range Lueyang black-bone chickens were selected as the research subjects, employing non-targeted metabolomics and microbiomics to analyze the distribution of antibiotics in intestinal tissues (duodenum and caecum) and muscle tissues (breast and leg muscles), and their correlations with the intestinal microbiota. Metabolomics detected 47 antibiotics in intestinal tissues and 22 in muscle tissues, with 9 common to both tissues, including clinically and veterinary relevant antibiotics such as oxacillin, kanamycin, and tobramycin. Microbiomics analysis indicated significant differences in microbial communities between the duodenum and caecum at the genus level. LEfSe analysis identified seven characteristic genera in the duodenum (e.g., *Bacteroides*, *Alistipes*) and five in the caecum (e.g., *Lactobacillus*, *Ureaplasma*). Pearson correlation analysis further revealed that these shared antibiotics were significantly associated with the differential genera in the intestine. For instance, oxacillin exhibited a positive correlation with both *Bacteroides* and *Alistipes*. Kanamycin was positively correlated with *Alistipes*, whereas tobramycin showed a negative correlation with *Bacteroides*. These results indicate that antibiotic residues were present in both intestinal and muscle tissues of Lueyang black-bone chickens raised under free-range mountainous conditions. The nine antibiotics common to both tissues are likely absorbed in the intestines and transported to muscles via the bloodstream. It is hypothesized that the gut microbiota may play a potential regulatory role in this process, providing a theoretical basis for understanding microecological mechanisms under environmental antibiotic exposure.

## 1. Introduction

Since the 1950s, antibiotics have been extensively used in animal husbandry for growth promotion, disease control, and infection prevention [1]. The majority are administered as feed additives at subtherapeutic doses to enhance feed conversion and growth efficiency, primarily through modulation of the gut microbiota [1,2]. However, the prolonged use of antibiotics has led to their persistent accumulation in the environment [3]. Approximately 30–90% of the antibiotics ingested by animals are excreted through urine and feces, either in their original form or as metabolites, subsequently entering soil and water bodies and causing environmental contamination [4,5]. Residual antibiotics in these habitats promote the proliferation of antibiotic-resistant bacteria (ARBs) and the horizontal transfer of antibiotic resistance genes (ARGs), which may spread through the food chain and water sources, ultimately posing significant threats to ecosystem stability and human health [6].

Studies have shown that antibiotic residues in livestock products have long been a major concern affecting their safety [7]. Oral antibiotics are typically absorbed through the intestinal tract of animals, with the extent of absorption being jointly influenced by their pharmacokinetic properties, the physiological characteristics of the tissues, and the drug delivery system [8,9]. The duodenum, as the initial segment of the small intestine, plays a central role in antibiotic absorption due to its submucosa enriched with a dense capillary network, the extensive absorptive surface area of epithelial cells, and the abundant expression of transporters [10,11]. In contrast, the caecum, located at the junction of the small and large intestines, primarily facilitates the passive diffusion of water and electrolytes. Its relatively limited mucosal surface area and absorptive capacity make it significantly less efficient for drug absorption compared to the duodenum [12,13]. After absorption, antibiotics first enter the liver via the portal vein, where some are metabolized and degraded, while the others that are not metabolized enter the bloodstream and are subsequently distributed to tissues throughout the body, leading to residues [14]. Studies have shown that antibiotic residues have been detected in the meat, eggs, and milk of livestock and poultry. Notably, higher detection rates have been reported in goat and cow milk, whereas comparatively lower levels are observed in eggs and chicken meat [15,16,17]. This difference may be related to factors such as the animal growth cycle, environmental conditions, and farming practices. Especially in intensive farming systems, the frequency and dosage of antibiotics are typically higher than those in free-range farming systems, owing to the high density of animal rearing and the greater risk of disease transmission. For instance, a study conducted on Spanish pig farms reported that the total antibiotic use in intensive farms (254.83 mg/PCU) was significantly higher than in organic/extensive farms (16.29 mg/PCU) [18]. Elevated antibiotic usage is generally associated with an increased risk of residues. Therefore, farming practices play a significant role in determining the levels of antibiotic residues in animal-derived products.

Furthermore, variations in microbiota composition across intestinal sites are also important factors influencing the absorption and tissue distribution of antibiotics. Significant differences in microbial diversity and metabolic functions between the duodenum and caecum of broilers have been demonstrated in previous studies [19]. In general, the duodenal microbiota exhibits low diversity, primarily dominated by facultative anaerobes, such as *Lactobacillus* and *Streptococcus*, whose low density, weakly acidic environment, and rapid transit properties limit microbial metabolic activity [20,21]. This may result in weak drug metabolism ability. In contrast, the caecal microbiota exhibits considerably higher diversity, dominated by obligate anaerobes, including members of the *Firmicutes* and *Bacteroidetes* phyla, with robust capabilities for polysaccharide, protein, and drug metabolism, and a prolonged retention time of caecal contents, which facilitates complex metabolic processes [22,23,24]. This microbiota difference can influence the efficiency of antibiotic absorption and the patterns of tissue distribution via multiple pathways. For example, antibiotic structures can be directly modified by gut microbiota through the secretion of metabolic enzymes, including hydrolases, lyases, oxidoreductases, and transferases, thereby altering their biological activities [25]. Additionally, intestinal flora can regulate the expression of drug metabolism enzymes and transporters in the host liver and intestine through their metabolites, such as short-chain fatty acids (SCFAs) and secondary bile acids, thereby changing the absorption efficiency of antibiotics and indirectly affecting the distribution of antibiotics [26]. Notably, the imbalance of intestinal flora can lead to the downregulation of tight junction protein expression in intestinal epithelial cells, increase intestinal permeability, and promote the entry of lipopolysaccharides (LPS) into the bloodstream [27]. LPS primarily binds to TLR4 and activates the NF-κB pathway, which induces the release of pro-inflammatory cytokines, such as IL-6 and TNF-α, thereby increasing the permeability of the vascular endothelial barrier [28]. Theoretically, increased vascular permeability may facilitate the diffusion of antibiotics from the blood circulation into peripheral tissues. However, due to the significant differences in the composition of individual microflora and the heterogeneity of the structure and blood flow distribution of muscle tissue itself [29,30,31], it is still a scientific hypothesis that the intestinal microflora affects the specific effect and degree of antibiotic distribution in muscle tissue through the above mechanism, which needs to be further verified.

Local Chinese chicken breeds, rich in germplasm resources, are traditionally raised in mountainous regions, resulting in excellent meat quality and distinctive flavor [32]. So, if antibiotics are not added in this traditional farming process, will antibiotic residues still be present in the chicken meat? Furthermore, is there any relationship between these residues and the gut microbiota? These questions remain unclear at present. Lueyang black-bone chicken is a unique local breed in China, traditionally raised through free-range farming in mountainous areas, and serves as an ideal model for studying this scientific issue. Therefore, this study focuses on Lueyang black-bone chicken to analyze the distribution of antibiotics in the intestinal (duodenum and caecum) and muscle tissues (breast and leg muscles), screen antibiotics shared by both the gut and muscles, and conduct an association analysis between these shared antibiotics and the gut microbiota. This study provides a theoretical foundation for establishing healthier breeding standards for Lueyang black-bone chickens.

## 2. Materials and Methods

### 2.1. Animal Ethics Statement

All animal experiments were performed in strict compliance with the ethical guidelines established by the Institutional Animal Care and Use Committee of Shaanxi University of Technology (2025031803).

### 2.2. Animal Resources and Breeding Systems

The experiment was conducted with 300 one-day-old healthy female Lueyang black-bone chickens. Following a 7 week domestication period in a uniform nesting environment, all birds were raised in a free-range system without grouping. Three replicates were established, each consisting of 100 chickens. The chickens were housed in an indoor bungalow (bird/0.15 m^2^) and an adjacent outdoor cage-free area (bird/1 m^2^). The indoor environmental conditions were controlled at an average temperature of 22–25 °C, relative humidity of 65–70%, and a photoperiod of 16 h light and 8 h dark. At the beginning of the formal experimental period, the average outdoor temperature in the local mountainous region was 22–25 °C. All chickens were supplemented with small amounts of local staple feed and green forage in the morning and evening, with free access to food and water at all times. After 20 weeks in the rearing system, the chickens were euthanized through carbon dioxide anesthesia, followed by routine neck cutting. It should be noted that systematic sampling and analysis of water, soil, and feed ingredients were not conducted in this study, which may limit the evaluation of the potential influence of environmental factors on the metabolomic and microbiomic outcomes.

### 2.3. Metabolomic Analysis of Intestinal and Muscle Tissues

After slaughter, intestinal (duodenum and caecum) and muscle (breast and leg muscles) tissues were isolated from six randomly selected chickens, immediately frozen in liquid nitrogen, and stored at −80 °C for subsequent metabolite analysis. The duodenum and caecum in the intestine were designated as Du and Ca, respectively, while the breast and leg muscles were referred to as BM and LM, respectively. Each treatment group included six biological replicates. Approximately 50 mg of intestinal and muscle tissue samples were placed in a 2 mL centrifuge tube along with a 6 mm diameter grinding bead. A total of 400 μL of extraction solution (methanol: water, 4:1, *v*/*v*) containing 0.02 mg/mL L-2-chlorophenylalanine as an internal standard was added. The samples were subjected to grinding for 6 min (−10 °C, 50 Hz), followed by low-temperature ultrasonic extraction (5 °C, 40 kHz) for 30 min. Subsequently, the extracts were incubated at −20 °C for 30 min and centrifuged at 13,000× *g* for 15 min at 4 °C. The resulting supernatants were collected for liquid chromatography tandem mass spectrometry (LC-MS/MS) analysis. A total of 20 µL of supernatant from each test sample was pooled and used as a quality control (QC) sample. A Thermo UHPLC-Q Exactive HF-X system equipped with an ACQUITY HSS T3 column (100 mm × 2.1 mm i.d., 1.8 μm; Waters, Milford, CT, USA) at Majorbio Bio-Pharm Technology Co., Ltd. (Shanghai, China) was used for separation and entered into mass spectrometry. The mobile phases consisted of solvent A (0.1% formic acid in water: acetonitrile, 95:5, *v*/*v*) and solvent B (0.1% formic acid in acetonitrile/isopropanol/water, 47.5:47.5:5, *v*/*v*). The injection volume was set at 3 μL, with a flow rate of 0.40 mL/min, and the column temperature was maintained at 40 °C. The mass spectrometric data were acquired using a Thermo UHPLC-Q Exactive HF-X Mass Spectrometer (Thermo Fisher Scientific, Waltham, MA, USA), equipped with an electrospray ionization (ESI) source in data-dependent acquisition (DDA) mode, covering a mass detection range of 70–1050 *m*/*z*.

Upon completion of mass spectrometry, the raw data were preprocessed using ProgenesisQI v3.0 software (Waters, Milford, CT, USA). During preprocessing, the internal standard peaks and all known false positive signals were removed from the data matrix, followed by redundant information filtering and peak merging. The metabolites were subsequently retrieved and identified through database searches. The processed data were uploaded to the Majorbio cloud platform (https://www.majorbio.com/, accessed on 10 March 2025) for further analysis. The response intensities of mass spectrum peaks in the samples were normalized to generate a standardized data matrix. The variables from QC samples with a relative standard deviation (RSD) > 30% were eliminated, and log10 logarithmically transformed to obtain the final data matrix for subsequent analysis. Then, principal component analysis (PCA) and partial least squares discrimination analysis (PLS-DA) were conducted using the R package “ropls” (version 1.6.2), and the stability of the model was evaluated by 7 cycle interactive validation. The metabolites with VIP > 1 and *p* < 0.05 were identified as significantly different, based on the variable importance in projection (VIP) scores derived from the PLS-DA model and the *p*-values derived from Student’s *t*-test.

### 2.4. The 16S rDNA Analysis of Gut Microbes

Bacterial genomic DNA was extracted from duodenal and caecal contents using the FastPure Stool DNA Isolation Kit (MP Biomedicals, Irvine, CA, USA). The quality and concentration of DNA were assessed via 1.0% agarose gel electrophoresis and a NanoDrop^®^ ND-2000 spectrophotometer (Thermo Fisher Scientific, Wilmington, DE, USA). The V3–V4 variable regions of the bacterial 16S rRNA gene were amplified with primer pairs 338F (5′-ACTCCTACGGGAGGCAGCAG-3′) and 806R (5′-GGACTACHVGGGTWTCTAAT-3′) by an ABI GeneAmp^®^ 9700 PCR thermocycler (Thermo Fisher Scientific, Wilmington, DE, USA). All samples were performed according to the formal experimental conditions, with three replicates for each sample. The PCR reaction mixture consisted of 10 μL 2×Pro Taq, 0.8 μL each primer (5 μM), 10 ng of template DNA, and ddH_2_O to a final volume of 20 µL. The PCR product was extracted from 2% agarose gel and purified, then quantified using Quantus™ Fluorometer (Promega, Madison, WI, USA). Purified amplicons were pooled in equimolar amounts and paired-end sequenced on an Illumina PE300 platform (Illumina, San Diego, CA, USA) according to the standard protocols by Majorbio Bio-Pharm Technology Co., Ltd. (Shanghai, China). The optimized data were processed using a sequence denoising method (DADA2/Deblur) to obtain the representative amplicon sequence variants (ASVs) and abundance information. Taxonomic classification, species richness and diversity analysis, differential abundance analysis, and microbial functional prediction were performed on the Majorbio cloud platform (https://www.majorbio.com/). Notably, taxonomy based on 16S rRNA gene V3–V4 region sequencing is generally reliable at the genus level, with certain taxa resolvable to the species level. However, its resolution remains limited for accurate species-level identification across all microorganisms. Furthermore, this approach relies on the completeness and accuracy of reference databases, and its functional predictions require validation through complementary omics analyses or targeted experimental methods.

### 2.5. Statistical Analysis

All data are expressed as mean ± standard deviation (SD) (*n* = 6). One-way analysis of variance (ANOVA) was conducted using GraphPad Prism (version 8.0.2). All parameters were repeated three times. A *p*-value < 0.05 was considered statistically significant. In addition, Pearson correlation analysis was performed to assess the relationship between metabolites and microbial communities. Significance levels were defined as follows * 0.01 < *p* ≤ 0.05, ** 0.001 < *p* ≤ 0.01, and *** *p* ≤ 0.001.

## 3. Results

### 3.1. Gut Metabolome Analysis

#### 3.1.1. Multivariate Statistical Analysis of Intestinal Metabolites

**Figure 1:**
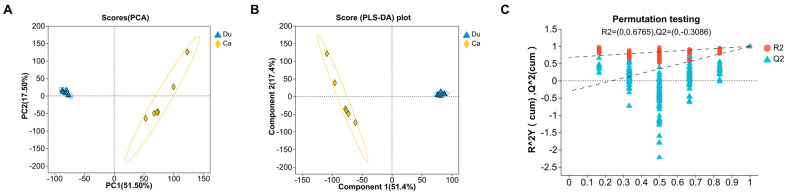
Multivariate analysis of metabolomic data from different intestinal samples. (**A**) Scatter plot of metabolites based on PCA in combined positive and negative ion modes. (**B**) Scatter plot of metabolites based on PLS-DA in combined positive and negative ion modes. (**C**) Scatter plot based on PLS-DA permutation test in combined positive and negative ion modes; the dashed lines represent the regression lines for the R2 and Q2 intercepts.

Metabolomics technology has been extensively applied in livestock and poultry breeding research, with LC-MS/MS analysis used to identify metabolites in samples. To highlight the distribution trends of intestinal metabolomics data and the degree of difference between groups, PCA and PLS-DA were performed in combined positive and negative ion modes. The results of PCA and PLS-DA showed that both groups of samples could be clearly separated, indicating significant differences in metabolites across different intestinal sites (Figure 1A,B). To avoid model overfitting, 200 permutation tests were conducted on the PLS-DA model. The validation of the PLS-DA model revealed that the intercept of the Q2 regression line on the *y*-axis was less than zero, suggesting that the model was well-fitted and exhibited high predictive capability (Figure 1C). Overall, the multivariate statistical analyses indicated that the intestinal metabolomics data were suitable for further data analysis.

#### 3.1.2. Analysis of Antibiotics in Gut Metabolites

**Figure 2:**
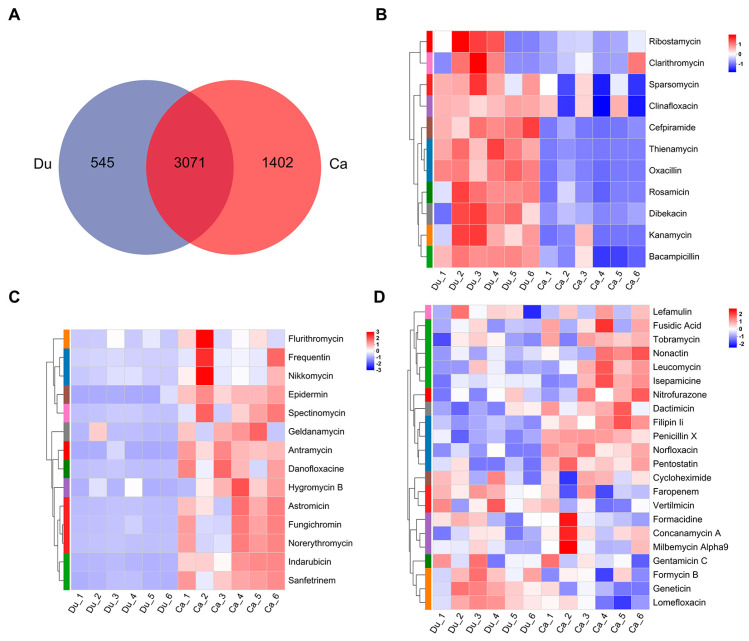
Analysis of antibiotics in intestinal metabolites. (**A**) Venn diagram of metabolites detected in the duodenum and caecum. (**B**) Hierarchical clustering heatmap showing the 11 antibiotics unique to the duodenum: ribostamycin, clarithromycin, sparsomycin, clinafloxacin, cefpiramide, thienamycin, oxacillin, rosamicin, dibekacin, kanamycin, and bacampicillin. (**C**) Hierarchical clustering heatmap showing the 14 antibiotics unique to the caecum: flurithromycin, frequentin, nikkomycin, epidermin, spectinomycin, geldanamycin, danofloxacin, antramycin, hygromycin B, astromicin, fungichromin, norerythromycin, indarubicin, and sanfetrinem. (**D**) Hierarchical clustering heatmap of the 22 antibiotics shared between the duodenum and caecum. Six had higher relative abundances in the duodenum (formycin B, geneticin, lomefloxacin, gentamicin C, vertilmicin, and faropenem), while sixteen were more abundant in the caecum (lefamulin, fusidic acid, tobramycin, nonactin, leucomycin, isepamicin, nitrofurazone, cycloheximide, formacidine, concanamycin A, milbemycin Alpha9, filipin II, penicillin X, norfloxacin, pentostatin, and dactimicin). The colored bands adjacent to the antibiotic names in heatmaps (**B**–**D**) represent distinct clusters identified by hierarchical clustering.

To investigate the distribution patterns of antibiotics in the duodenum and caecum of Lueyang black-bone chickens, metabolites from both intestinal regions were analyzed. A total of 5018 metabolites were identified in the duodenum and caecum. Of these, 545 metabolites were found exclusively in the duodenum, 1402 were unique to the caecum, and 3071 were shared by both intestinal segments (Figure 2A). This suggests that the caecum contains a greater diversity of metabolites than the duodenum, while also indicating similarities in certain metabolic pathways between the two sites. Among the metabolites unique to the duodenum, 11 antibiotics were identified: ribostamycin, clarithromycin, sparsomycin, clinafloxacin, cefpiramide, thienamycin, oxacillin, rosamicin, dibekacin, kanamycin, and bacampicillin (Figure 2B). In contrast, 14 antibiotics were found only in the caecum: flurithromycin, frequentin, nikkomycin, epidermin, spectinomycin, geldanamycin, danofloxacin, antramycin, hygromycin B, astromicin, fungichromin, norerythromycin, indarubicin, and sanfetrinem (Figure 2C). In addition, 22 antibiotics were detected in both intestinal segments. Of these, six (formycin B, geneticin, lomefloxacin, gentamicin C, vertilmicin, and faropenem) showed higher relative abundance in the duodenum, whereas sixteen (lefamulin, fusidic acid, tobramycin, nonactin, leucomycin, isepamicin, nitrofurazone, cycloheximide, formacidine, concanamycin A, milbemycin Alpha9, filipin II, penicillin X, norfloxacin, pentostatin, and dactimicin) were more abundant in the caecum (Figure 2D). Overall, these findings reveal distinct differences in antibiotic composition and relative abundance between the duodenum and caecum. Each intestinal site possesses characteristic antibiotics with high expression levels, likely reflecting their specific physiological functions and microbial environments.

### 3.2. Muscle Metabolome Analysis

#### 3.2.1. Multivariate Statistical Analysis of Muscle Metabolites

**Figure 3:**
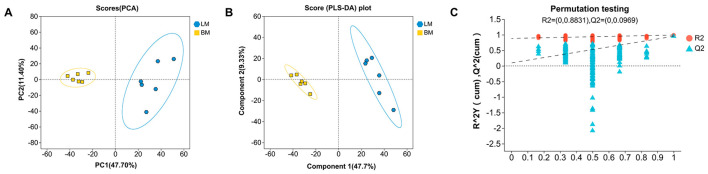
Multivariate analysis of metabolomic data from different muscle samples. (**A**) Scatter plot of metabolites based on PCA in combined positive and negative ion modes. (**B**) Scatter plot of metabolites based on PLS-DA in combined positive and negative ion modes. (**C**) Scatter plot based on PLS-DA permutation test in combined positive and negative ion modes; the dashed lines represent the regression lines for the R2 and Q2 intercepts.

To highlight the distribution trends of muscle metabolomics data and the degree of difference between groups, PCA and PLS-DA were used for analysis in combined positive and negative ion modes. The results of PCA and PLS-DA analysis showed that both groups of samples could be clearly separated, indicating significant differences in metabolites across different muscle tissues (Figure 3A,B). To prevent model overfitting, 200 permutation tests were conducted on the PLS-DA model. The validation of the PLS-DA model showed that both the R2 and Q2 values declined with decreasing permutation retention, while the regression line exhibited an upward trend, confirming that the permutation test passed and the model was not overfit (Figure 3C). Multivariate statistical analysis showed the suitability of muscle metabolomics data for subsequent analysis.

#### 3.2.2. Analysis of Antibiotics in Muscle Metabolites

**Figure 4:**
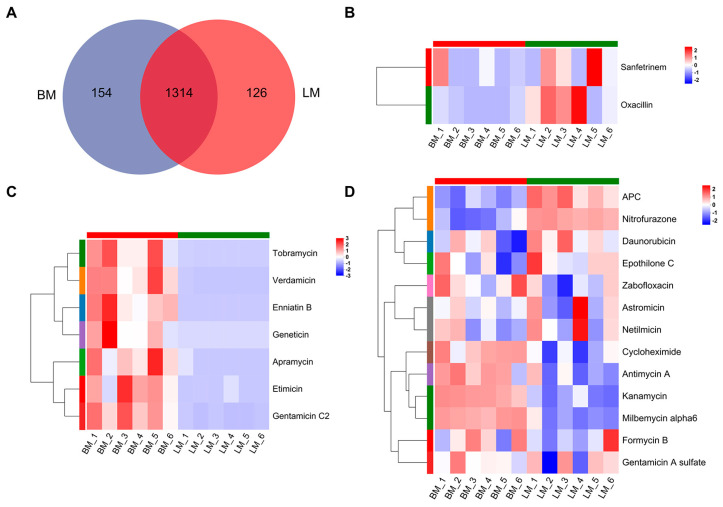
Analysis of antibiotics in muscle metabolites. (**A**) Venn diagram of metabolites detected in the breast and leg muscles. (**B**) Hierarchical clustering heatmap showing the two antibiotics unique to the leg muscle: oxacillin and sanfetrinem. (**C**) Hierarchical clustering heatmap showing the seven antibiotics unique to the breast muscle: enniatin B, geneticin, tobramycin, apramycin, etimicin, verdamicin, and gentamicin C2. (**D**) Hierarchical clustering heatmap of the 13 antibiotics shared between the breast and leg muscles. Seven exhibited higher relative abundance in the breast muscle (zabofloxacin, cycloheximide, antimycin A, milbemycin alpha6, kanamycin, formycin B, and gentamicin A sulfate), whereas six were more abundant in the leg muscle (APC, nitrofurazone, epothilone C, daunorubicin, netilmicin, and astromicin). The colored bands adjacent to the antibiotic names in heatmaps (**B**–**D**) represent distinct clusters identified by hierarchical clustering.

To investigate the trends in antibiotic variation between the breast and leg muscles of Lueyang black-bone chickens, metabolites from both muscle tissues were analyzed. A total of 1594 metabolites were identified, of which 154 were unique to the breast muscle, 126 were exclusive to the leg muscle, and 1314 were shared between the two tissues (Figure 4A). The presence of shared metabolites suggests a high degree of similarity in metabolic pathways between the two muscle types. Within the metabolites detected exclusively in the leg muscle, two antibiotics were identified, namely, oxacillin and sanfetrinem (Figure 4B). In contrast, seven antibiotics were found only in the breast muscle, including enniatin B, geneticin, tobramycin, apramycin, etimicin, verdamicin, and gentamicin C2 (Figure 4C). Furthermore, thirteen antibiotics were present among the metabolites shared by both muscle types. Of these, seven antibiotics, including zabofloxacin, cycloheximide, antimycin A, milbemycin alpha6, kanamycin, formycin B, and gentamicin A sulfate, showed higher relative abundance in the breast muscle. The remaining six antibiotics, which were APC, nitrofurazone, epothilone C, daunorubicin, netilmicin, and astromicin, exhibited higher relative abundance in the leg muscle (Figure 4D). Overall, these results demonstrate clear differences in both the composition and expression levels of antibiotics between breast and leg muscles, reflecting variations in their metabolic characteristics and tissue-specific accumulation patterns.

### 3.3. Analysis of Antibiotics in the Gut and Muscle Tissues

**Figure 5:**
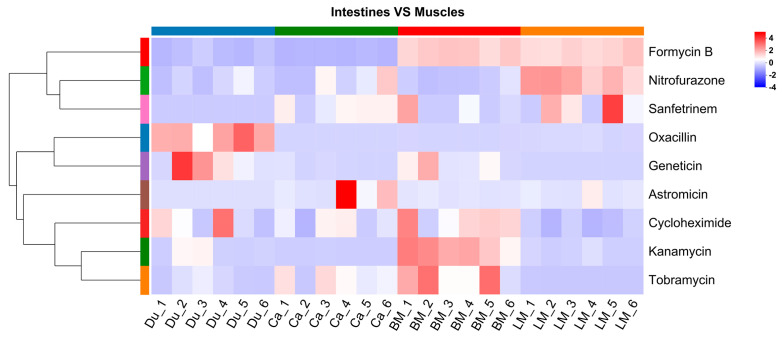
Hierarchical clustering of antibiotics shared among different intestinal tracts (duodenum and caecum) and muscle tissues (breast and leg muscles), including oxacillin, geneticin, kanamycin, tobramycin, cycloheximide, formycin B, astromicin, sanfetrinem, and nitrofurazone. The colored bands adjacent to the antibiotic names in heatmaps represent distinct clusters identified by hierarchical clustering.

A total of nine antibiotics were identified to be common to both intestinal and muscle tissues. Detailed detection sites and possible absorption routes for each are provided in Table S1. Overall, these data suggest that antibiotics may be absorbed in different regions of the intestine and subsequently distributed to specific muscle groups. Hierarchical clustering was performed, based on the antibiotic concentrations to analyze the variation trends of antibiotics in different intestinal and muscle tissues (Figure 5). The results showed that oxacillin and geneticin were relatively abundant in the duodenum. Kanamycin, tobramycin, cycloheximide, and formycin B exhibited relatively higher relative abundance in the pectoralis muscle. Astromicin displayed a higher relative abundance in the caecum. Sanfetrinem and nitrofurazone exhibited relatively higher abundance in the leg muscles. These results suggest that the distribution of antibiotics in different intestinal and muscle tissues is tissue specific.

### 3.4. Gut Microbiota Analysis

**Figure 6:**
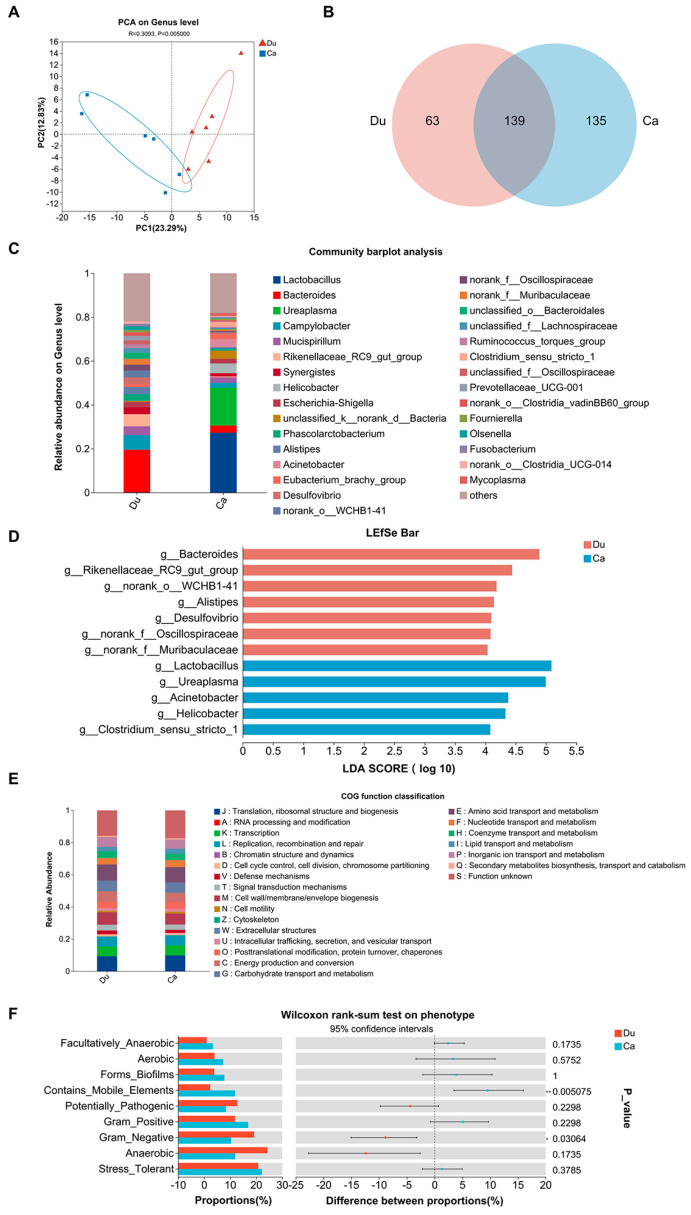
Genus-level composition and functional prediction analysis of gut microbes in the duodenum and caecum groups. (**A**) PCA plot. (**B**) Venn diagram showing shared and unique genera between groups. (**C**) Relative abundance of microbial community composition at the genus level. (**D**) Histogram of linear discriminant analysis (LDA) scores based on LEfSe analysis. (**E**) COG functional abundance profiles predicted by PICRUSt2. (**F**) Group difference test plot based on BugBase phenotype predictions (top 9 phenotypes; * 0.01 < *p* ≤ 0.05, ** 0.001 < *p* ≤ 0.01, *** *p* ≤ 0.001).

The optimized data were processed using the sequence denoising method (DADA2/Deblur), resulting in the identification of an average of 898 and 320 ASVs in the two groups (*p* > 0.05, Table 1). Good’s coverage was employed to assess whether the sequencing methods accurately characterized gut microbiota composition [33]. In this study, almost all sequences in both groups exhibited complete Good’s coverage (*p* > 0.05, Table 1). Although no significant differences were observed in the alpha diversity indices (Ace, Sobs, Chaol, Ace, Simpson, and Shannon) between the two groups, the richness of the gut microbiota was obviously higher in the duodenum than in the caecum (*p* > 0.05, Table 1). Furthermore, the PCA revealed significant differences in gut microbiota at the genus level between the two sample groups (*p* = 0.05, Figure 6A). At the genus level, the Venn diagram showed that 63 genera were unique to the duodenum, 135 genera were unique to the caecum, and 139 genera were shared (Figure 6B). Community composition analysis revealed that the three most dominant genera in the duodenum were *Bacteroides* (19.44%), *Campylobacter* (6.62%), and *Rikenellaceae_RC9_gut_group* (5.52%), whereas in the caecum, the dominant genera were *Lactobacillus* (27.27%), *Ureaplasma* (17.30%), and *Helicobacter* (4.46%) (Figure 6C). LEfSe analysis (*LDA* > 4, *p* < 0.05) identified seven genera as potential biomarkers of the duodenum: *Bacteroides*, *Rikenellaceae_RC9_gut_group*, *norank_o_WCHB1-41*, *Alistipes*, *Desulfovibrio*, *norank_f_Oscillospiraceae*, and *norank_f_Muribaculaceae*. Five genera were identified as potential biomarkers of the caecum: *Lactobacillus*, *Ureaplasma*, *Acinetobacter*, *Helicobacter*, and *Clostridium_sensu_stricto_1* (Figure 6D). COG functional prediction using PICRUSt2 indicated that functional categories such as amino acid transport and metabolism; cell wall/membrane/envelope biogenesis; and translation, ribosomal structure, and biogenesis were relatively abundant in both groups (Figure 6E). Additionally, BugBase phenotype prediction showed that Gram-negative bacteria were more abundant in the duodenum (*p* = 0.03064), whereas mobile element-containing bacteria were more abundant in the caecum (*p* = 0.005075) (Figure 6F). Overall, these results indicate that the duodenum and caecum harbor highly diverse microbial communities with distinct taxonomic compositions, biomarker genera, and functional potential.

### 3.5. Association Analysis Between Gut Microbiota and Antibiotics

**Figure 7:**
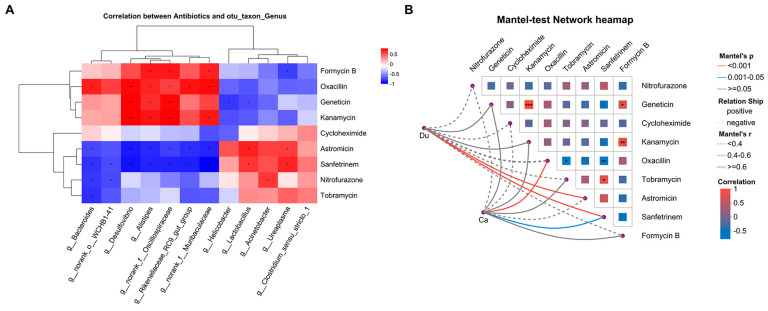
Pearson correlation analysis between antibiotics and gut microbial genera. (**A**) Heatmap of antibiotics and gut microbes, revealing the relationships between antibiotics and microbial genera. (**B**) A Mantel test network heatmap was constructed to evaluate the relationships between antibiotics and microbial genera in the duodenum and caecum (* 0.01 < *p* ≤ 0.05, ** 0.001 < *p* ≤ 0.01, *** *p* ≤ 0.001).

To further elucidate the relationships between shared antibiotics in the gut and muscle and the gut microbiota, Pearson correlation analysis at the genus level was performed between nine shared antibiotics and twelve differential bacterial genera (Figure 7A). Formycin B was positively correlated with *Alistipes*, *norank_f_Oscillospiraceae*, and *norank_f_Muribaculaceae* (*p* ≤ 0.05), while oxacillin showed positive correlations with *Bacteroides*, *Desulfovibrio*, *Alistipes*, *norank_f_Oscillospiraceae*, *Rikenellaceae_RC9_gut_group*, and *norank_f_Muribaculaceae* (*p* ≤ 0.05 or *p* ≤ 0.01). Geneticin and kanamycin were positively correlated with *Desulfovibrio*, *Alistipes*, and *norank_f_Oscillospiraceae* (*p* ≤ 0.05 or *p* ≤ 0.01), and kanamycin was also correlated with *norank_f_Muribaculaceae* (*p* ≤ 0.05). These antibiotics were more abundant in the caecum. Astromicin and sanfetrinem were negatively correlated with *Bacteroides*, *Desulfovibrio*, *Alistipes*, *norank_f_Oscillospiraceae*, and *Rikenellaceae_RC9_gut_group* (*p* ≤ 0.05 or *p* ≤ 0.01), with sanfetrinem also correlated with *norank_o_WCHB1-41* (*p* ≤ 0.05). Nitrofurazone was negatively correlated with *Bacteroides* and *norank_o_WCHB1-41* (*p* ≤ 0.05), and tobramycin with *Bacteroides* (*p* ≤ 0.05). These antibiotics were less abundant in the caecum. Conversely, astromicin and sanfetrinem were positively correlated with *Lactobacillus* and *Ureaplasma*, and nitrofurazone with *Acinetobacter* (*p* ≤ 0.05), showing higher abundance in the duodenum. Formycin B was negatively correlated with *Ureaplasma*, and geneticin with *Helicobacter* and *Lactobacillus* (*p* ≤ 0.05), showing lower abundance in the duodenum.

Additionally, Mantel test results (Figure 7B) indicated that the duodenal microbiota was significantly correlated with astromicin and sanfetrinem (*p* ≤ 0.001), whereas the caecal microbiota was correlated with oxacillin (*p* ≤ 0.001) and sanfetrinem (0.001 < *p* ≤ 0.05). These findings suggest that specific intestinal genera are closely associated with the distribution of these shared antibiotics.

## 4. Discussion

### 4.1. Analysis of Potential Sources of Antibiotic Tissue Residues in Lueyang Black-Bone Chickens

Antibiotic residues refer to the presence of incompletely metabolized or excreted antibiotic components in food products, animals, or the environment, posing significant threats to human health and contributing to the emergence of drug resistance. Although progress has been made in elucidating the mechanisms underlying antibiotic residues, effective environmental monitoring and intervention strategies remain lacking in antibiotic-free rearing systems. Therefore, a comprehensive understanding of the potential pathways of antibiotic residue formation in livestock and poultry is essential for developing precise ecological regulation strategies. In this study, Lueyang black-bone chickens were raised under an ecological stocking mode, where they freely foraged on natural feed, including insects and weeds, during the day; drank from natural water sources; and were supplemented with small amounts of local staple feed and green forage in the morning and evening. Under such conditions, environmental exposure is likely to constitute the primary source of antibiotic intake in chickens. Previous studies have shown that residual antibiotics in livestock manure can be absorbed by plant roots and enriched in crops after entering the soil through fertilizer application [34]. During foraging, poultry may ingest these plants or prey on insects that feed on them, thereby acquiring antibiotics indirectly. In addition, multiple antibiotics, including quinolones, tetracyclines, sulfonamides, and macrolides, have been frequently detected in untreated groundwater and surface water within areas of intensive livestock farming [35,36,37,38]. Although our study focused on free-range chickens in low-density ecosystems rather than intensive farms, these reports suggest that regional environmental reservoirs of antibiotics persist. Antibiotics from intensive agricultural areas may migrate through interconnected surface and groundwater networks, thereby posing a potential risk to the surrounding free-range environments [39,40]. Furthermore, several studies have reported that airborne particles and dust in farm environments harbor abundant ARGs and ARBs. These materials can be deposited into soil and water via wet and dry deposition, thereby establishing a circulating environmental exposure pathway [41,42,43]. Therefore, the antibiotics detected in the intestines (47 types) and muscle tissues (22 types) of Lueyang black-bone chickens in this study are most likely associated with environmental exposure. However, it should be noted that some identified antibiotics are relatively rare, and certain detected signals may represent degradation products, metabolites, or structural analogs from the environment rather than the parent compounds [44]. This issue is particularly significant in antibiotic-free rearing systems. Future investigations are encouraged to integrate targeted quantitative analysis with structural elucidation to confirm the chemical nature and sources of these signals and to strengthen systematic monitoring of environmental media such as water, soil, and insects.

### 4.2. Antibiotic Tissue Distribution Patterns and Absorption-Transport Characteristics

Metabolites in gut and muscle tissues provide abundant metabolic information and are strongly correlated with the intestinal microbiota. In this study, nine shared antibiotics were detected in different intestinal segments and muscle tissues by non-targeted metabolomics. Moreover, these antibiotics exhibited specific distribution patterns across the various intestinal and muscle tissues. Specifically, oxacillin is primarily distributed in the duodenum and leg muscles; kanamycin is detected in the duodenum, breast muscles, and leg muscles; sanfetrinem is found in the caecum and leg muscles; astromicin is present in the caecum, breast muscles, and leg muscles; geneticin and tobramycin are simultaneously detected in the duodenum, caecum, and breast muscles; whereas nitrofurazone, formycin B, and cycloheximide are distributed across the duodenum, caecum, and both breast and leg muscles. The presence of these antibiotics in different intestinal and muscle tissues indicates that they may possess certain absorption and transport capabilities within the body. Existing studies have indicated that the absorption of orally administered drugs primarily occurs in the small intestine, particularly in the duodenum and jejunum [45]. The caecum, on the other hand, primarily functions in the absorption of water and electrolytes, with a weaker drug absorption capacity [12]. Additionally, the leg and breast muscles in this study also exhibited differences in antibiotic residues. In broiler chickens, the breast muscles are composed primarily of type II fast-twitch fibers, characterized by low mitochondrial density and a reliance on glycolytic metabolism. Conversely, the leg muscles are dominated by type I slow-twitch fibers, which are rich in mitochondria and primarily utilize oxidative phosphorylation for energy production [46]. These metabolic differences may explain the variation in antibiotic distribution between the two muscle types. Following absorption by intestinal epithelial cells, antibiotics are transported into the interstitial fluid via passive diffusion or basement membrane transporters, traverse the capillary walls, enter the circulatory system, and are subsequently distributed to tissues and organs throughout the body via the bloodstream [47,48]. Based on these results, we can speculate that oxacillin may be distributed to the leg muscles via duodenal absorption. Kanamycin may be delivered to both the breast and leg muscles through duodenal absorption. Sanfetrinem may be absorbed through the caecum and distributed to the leg muscles. Astromicin may be similarly distributed to the breast and leg muscles via caecum absorption. Additionally, geneticin and tobramycin may be transported to the breast muscles through both duodenal and caecal absorption, while nitrofurazone, formycin B, and cycloheximide may be distributed to the breast and leg muscles via both pathways.

### 4.3. Potential Regulatory Role of Intestinal Microbial Differences in Antibiotic Distribution

The gut microbiota, often referred to as the second-largest genome of the host, plays a critical role in maintaining normal physiological functions. Increasing evidence indicates significant differences in microbial diversity and metabolic function between the duodenum and caecum [19,49,50]. In this study, 16S rDNA amplicon sequencing was employed to analyze the gut microbial characteristics of the duodenum and caecum. The microbial compositions of the duodenum and caecum were found to differ significantly at the genus level, consistent with previous findings. Furthermore, LEfSe analysis identified 12 differential genera between the duodenum and caecum. *Bacteroides*, *Rikenellaceae_RC9_gut_group*, *norank_o_WCHB1-41*, *Alistipes*, *Desulfovibrio*, *norank_f_Oscillospiraceae*, and *norank_f_Muribaculaceae* were identified as biomarkers of the duodenum, whereas *Lactobacillus*, *Ureaplasma*, *Acinetobacter*, *Helicobacter*, and *Clostridium_sensu_stricto_1* were identified as biomarkers of the caecum. Firmicutes and Bacteroidetes, the two dominant phyla within the gut microbiota, have been found to participate in the production of SCFAs through the fermentation of dietary fiber and the decomposition of complex polysaccharides. SCFAs mainly consist of acetic acid, propionic acid, and butyric acid [51]. Among these, bacteria from the Firmicutes phylum predominantly produce butyric acid, whereas those from the Bacteroidetes phylum primarily secrete acetic and propionic acids [52]. Butyric acid serves as the primary energy source for colonic epithelial cells, and exhibits anti-inflammatory effects, strengthens the intestinal barrier, and regulates immune functions [53]. Acetate participates in lipid synthesis and plays a crucial role in maintaining energy balance and metabolic homeostasis [54]. Propionic acid contributes to metabolic and nervous system protection by activating PPARα, thereby promoting fatty acid oxidation, inhibiting inflammatory pathways, and safeguarding the integrity of the blood–brain barrier [55,56]. For example, Rikenellaceae_RC9_gut_group, Alistipes, Muribaculaceae, and Bacteroides in this study belong to the phylum Bacteroidetes, and are major producers of acetic and propionic acids [57,58,59,60]. Oscillospiraceae belongs to the phylum Firmicutes and predominantly produces butyric acid [61]. Additionally, our study also found that the number of Gram-negative bacteria is higher in the duodenum, while the caecum harbors a greater number of microbiota carrying mobile genetic elements (MGEs). Previous studies have pointed out a significant positive correlation between ARGs and MGEs in the chicken gut, with intestinal segments having a higher abundance of ARGs, often accompanied by more MGEs [62]. These findings suggest that the duodenum may promote efficient nutrient absorption by enriching Gram-negative polysaccharide-degrading microbiota, while the caecum is more likely to accumulate anaerobic fermentative bacteria and potential pathogens carrying MGEs, adapting to its complex fermentation environment. This could facilitate the spread of antibiotic resistance and contribute to the onset of chronic inflammatory responses.

Furthermore, correlations between twelve bacterial genera and nine commonly used antibiotics across intestinal and muscle tissues were further analyzed. Significant associations were observed between formycin B and *Alistipes*, *norank_f__Oscillospiraceae*, and *norank_f__Muribaculaceae.* Oxacillin, geneticin, and kanamycin were correlated with *Alistipes* and *norank_f__Oscillospiraceae*, and geneticin was additionally associated with *Lactobacillus*. Astromicin and sanfetrinem showed significant correlations with multiple genera, including *Bacteroides*, *Alistipes*, *norank_f__Oscillospiraceae*, *Rikenellaceae_RC9_gut_group*, and *norank_f__Muribaculaceae.* Nitrofurazone was significantly associated with *Bacteroides* and *Acinetobacter*, while tobramycin was significantly associated with *Bacteroides*. From the perspective of molecular mechanism, Formycin B, a nucleoside antibiotic, is converted into formycin B 5′-monophosphate by nucleoside phosphotransferase, subsequently producing a triphosphate derivative of Formycin A that is incorporated into RNA, thereby interfering with nucleic acid metabolism and inducing cytotoxicity [63]. The transmembrane transport of formycin B is primarily mediated by sodium-dependent nucleoside transporters located on the cell membrane [64]. Formycin B remains neutral at pH 7 but becomes ionized under extreme pH conditions [65]. Oxacillin, a β-lactamase-resistant penicillin derivative, possesses side chain modifications that confer high resistance to β-lactamase-mediated degradation [66]. Therefore, oxacillin is generally not efficiently hydrolyzed, even in strains capable of producing penicillinase. Notably, oxacillin exhibits an oral bioavailability of 25–73% and a plasma protein binding rate of 74–97% [67,68]. This may restrict its diffusion into tissues. Furthermore, the minimum inhibitory concentration (MIC) of oxacillin has been reported to decrease significantly as the pH drops from 7.4 to 5.0, indicating that acidic environments may enhance its antimicrobial efficacy [69]. Sanfetrinem, a tricyclic β-lactam antibiotic, exhibits enhanced stability against β-lactamase degradation, due to its tricyclic structural design [70]. The oral bioavailability of sanfetrinem, administered as the prodrug sanfetrinem cilexetil, was reported to be 32% in rats and 15% in dogs [71]. This suggests that there is some degree of systemic absorption. Astromicin, tobramycin, kanamycin, and geneticin are classified as aminoglycoside antibiotics [72,73,74]. Aminoglycosides, characterized by their polarity due to multiple amino and hydroxyl groups, are generally poorly absorbed when administered orally. Due to their high polarity, these antibiotics are poorly lipid-soluble and, therefore, unable to efficiently cross the lipid bilayer via passive diffusion [75]. For example, the oral absorption rate of tobramycin ranges from 23% to 47% [76]. In this study, these antibiotics were still detected in the muscles, suggesting that the intestinal environment may facilitate their absorption to some extent. In addition, the passive diffusion of nitrofurazone into the body depends on concentration gradients and the permeability of the intestinal epithelium, occurring via paracellular or transcellular pathways [77,78,79]. When the integrity of the intestinal barrier is compromised, intercellular tight junctions are loosened, leading to increased epithelial permeability and enhanced passive diffusion efficiency of the drug [80]. Moreover, the solubility of nitrofurazone is extremely low under neutral to mildly acidic conditions (pH 5.0–7.5), whereas its degradation rate markedly increases at approximately pH 5.0 [81]. It suggests that the bioavailability of nitrofurazone may be influenced more by promoting its degradation rather than by altering its solubility. Based on this, some gut microbiota identified in this study (e.g., *Alistipes*, *Rikenellaceae_RC9_gut_group*, *Bacteroides*, *Lactobacillus*, and *Acinetobacter*) may indirectly regulate the intestinal environment and barrier function through their metabolites, such as SCFAs, thereby influencing antibiotic distribution in muscle tissues.

Geneticin resistance is mediated by genes such as neo, which encodes aminoglycoside phosphotransferase (APH(3′)II). This enzyme inactivates geneticin through phosphorylation [82]. *Lactobacillus* typically exhibits inherent resistance to aminoglycoside antibiotics due to the absence of a cytochrome-mediated electron transport system in its cell membrane, resulting in the inability of the active transport of aminoglycosides into the cell [83]. Furthermore, the substrate specificity of aminoglycoside phosphotransferase is highly selective; current studies have not detected neo-encoded APH(3′)II in *Lactobacillus* [84]. Studies have demonstrated that even in the absence of acquired resistance genes, the MIC of *Lactobacillus* against aminoglycosides is significantly higher than that of other bacteria, indicating that its inherent resistance is dominant [85]. Therefore, the negative correlation observed between *Lactobacillus* and geneticin in this study is more likely mediated by non-metabolic mechanisms, such as drug efflux and decreased membrane permeability, rather than by direct metabolic effects of the bacterium. Nitrofurazone, a broad-spectrum nitrofuran antibiotic, undergoes stepwise NADPH-dependent reduction in its nitro group catalyzed by nitroreductase enzymes, resulting in the formation of hydroxylamine and amino derivatives that subsequently generate stable tissue-bound metabolites, including semicarbazone and semicarbazide [86]. Previous research has confirmed that some intestinal microbiota can promote the metabolism of nitrofurazone via nitroreductase activity [78]. *Acinetobacter* is a Gram-negative bacterium that preferentially colonizes aerobic environments with limited nutrient availability [87]. Its strains are capable of acquiring multidrug resistance through mechanisms such as gene mutation, horizontal gene transfer, efflux pumps, and enzymatic degradation [88]. Moreover, certain *Acinetobacter* strains have been demonstrated to possess nitroreductase activity, which enables the reduction in nitro groups in aromatic and heterocyclic compounds [89,90]. Therefore, *Acinetobacter* may directly participate in the decomposition and transformation of nitrofurazone through the nitroreductase system in this study, thereby gaining metabolic advantages and resistance characteristics in a specific microenvironment, potentially influencing its distribution in muscle tissues. It is important to note that this study relied solely on correlation analysis, and while the results suggest a potential link between gut microbiota and antibiotic distribution in muscle, they are insufficient to establish causality. Therefore, we propose that this association might indicate a possible pathway for microecological regulation between the gut and muscle.

## 5. Conclusions

This study revealed that, even under free-range conditions without artificial antibiotic supplementation, residues of 47 and 22 antibiotics were unexpectedly detected in the intestinal and muscle tissues of Lueyang black-bone chickens, respectively. The accumulation of antibiotics in free-range chickens is likely due to long-term environmental exposure, including contact with contaminated soil and water, consumption of naturally foraged plants and insects, and inhalation or contact with air and dust containing ARGs and ARBs. Notably, nine antibiotics were detected in both intestinal and muscle tissues and exhibited significant correlations with specific bacterial genera. These findings raise the possibility that environmental antibiotics reach muscle tissue through microbiota-associated pathways, although the underlying mechanisms remain to be elucidated. Future studies should incorporate environmental antibiotic monitoring, metagenomic approaches, and integrated multi-omics analyses, complemented by in vitro and in vivo functional validations, to elucidate specific microbial roles and to develop targeted microecological strategies for controlling antibiotic residues in poultry farming.

## Figures and Tables

**Table 1:** Diversity of the intestinal microflora of Lueyang black-bone chickens (*n* = 6).

Groups		Du	Ca
ASVs		898 ± 280.90	320 ± 141.82
Good’s coverage		0.9999 ± 0.0001	0.9999 ± 0.0001
Richness	Sobs	128.67 ± 12.75	114.5 ± 40.68
	Chaol	129.90 ± 12.89	116.24 ± 41.42
	Ace	130.08 ± 12.80	116.16 ± 41.01
Diversity	Shannon	3.27 ± 0.35	2.40 ± 0.90
	Simpson	0.09 ± 0.07	0.28 ± 0.22

## Data Availability

The original contributions presented in this study are included in the article. Further inquiries can be directed to the corresponding authors.

## Supplementary Materials

The following supporting information can be downloaded at: https://www.mdpi.com/article/10.3390/microorganisms13102239/s1, Table S1. Summary of the nine antibiotics detected in both intestinal and muscle tissues, their detection sites, and possible routes of absorption.
