# Supplementary material for: Antibiotic Residues in Muscle Tissues of Lueyang Black-Bone Chickens Under Free-Range Mountainous Conditions and Their Association with Gut Microbiota
Source: Microorganisms. 2025 Sep 24;13(10):2239. doi: 10.3390/microorganisms13102239 (PMC12566076; doi:10.3390/microorganisms13102239)
Supplement: Supplementary file 1 [file microorganisms-13-02239-s001.zip › microorganisms-3845339-supplementary.pdf]

Supplementary Materials

Table S1. Summary of the nine antibiotics detected in both intestinal and muscle tissues, their detection sites, and possible routes of absorption.

**Antibiotic**	**Detection Sites (Intestinal; Muscle)**	**Possible Route of Absorption**
Oxacillin	Duodenum; Leg	Via Duodenum to Leg muscle
Kanamycin	Duodenum; Breast; Leg	Via Duodenum to Breast and Leg muscles
Sanfetrinem	Caecum; Leg	Via Caecum to Leg muscle
Astromicin	Caecum; Breast; Leg	Via Caecum to Breast and Leg muscles
Geneticin	Duodenum; Caecum; Breast	Via Duodenum and Caecum to Breast muscle
Tobramycin	Duodenum; Caecum; Breast	Via Duodenum and Caecum to Breast muscle
Nitrofurazone	Duodenum; Caecum; Breast; Leg	Via Duodenum and Caecum to Multiple muscles
Formycin B	Duodenum; Caecum; Breast; Leg	Via Duodenum and Caecum to Multiple muscles
Cycloheximide	Duodenum; Caecum; Breast; Leg	Via Duodenum and Caecum to Multiple muscles
